# Patient eligibility criteria for a surgical treatment that enhances tissue sealing by use of a medicated sponge: observational study ELITE

**DOI:** 10.1186/2193-1801-2-613

**Published:** 2013-11-18

**Authors:** Cyril Raiffort, Farid Benabdallah, Jean-Louis Paillasseur, Yves Gangner, Eric Leutenegger, Philippe Sockeel

**Affiliations:** General and Gynaecologic Surgery, University Hospital of Lariboisière, 2 rue Amboise Paré, 75475 Paris, Cedex 10, France; Medical Affairs Direction, Takeda SAS France, Immeuble Pacific, 11-13 cours Valmy, 92977 Paris la Défense, France; Effi-Stat, 22 rue du Pont Neuf, 75001 Paris, France; Digestive Surgery Department, Hospital of Niort, 40 avenue Charles de Gaulle, 79021 Niort Cedex, France; ABR Pharma, 15 rue de Turbigo, 75002 Paris, France; HIA Legouest, 27 avenue des Plantières, 57077 Metz, Cedex 03, France

## Abstract

**Rationale:**

The application of a haemostatic agent in general surgery, in addition to its effect on bleeding, also provides tissue sealing and adhesion. A sponge drug is used with some actions of resection and wide dissection, without knowledge of the eligibility of patients. In this study, we sought to identify clusters of patients for which the medicated sponge enhances tissue sealing.

**Methods:**

Observational study (ELITE), from a panel of selected surgeons from hospitals in France in several surgical areas: abdominal, gynaecology, urology and thoracic. The survey identified the criteria for using the sponge TachoSil® in their surgical practices involving n = 683 patients. A multiple correspondence analysis (MCA) followed by an ascending hierarchical classification (AHC) was used in order to identify the eligibility criteria for the application of the sponge for tissue sealing in addition to hemostasis.

**Results:**

The most relevant classification was based on 9 groups of patients for which the sponge was used. 6 of them are mainly linked with the kind of lesion, 2 with the site of application and the latest one with the type of operation.

**Conclusions:**

The ELITE study revealed that the TachoSil® sponge was used mainly during surgery, requiring a reinforcement of the resection tissue sealing. The expected objective was successfully reached in 97% of the cases.

**Electronic supplementary material:**

The online version of this article (doi:10.1186/2193-1801-2-613) contains supplementary material, which is available to authorized users.

## Introduction

In all operative fields, hemostasis is a key element for determining a successful surgical outcome. Failure to achieve hemostasis contributes to high postoperative morbidity and mortality. The desire to manage hemostasis in an effective and minimally invasive manner has led to the development of surgical tissue sealing adhesives. In the early 1990s, fibrin sealants were combined with a collagen patch for use as a topical hemostasis agent, named Surgical Patch SP. Subsequent developments led to a third generation of SP which entailed complete removal of bovine aprotinin. Table 1 summarizes SP development from the first to the third generation.

**Table 1:** **SP development steps**

Product development		
**SP-1 (TachoComb®)**	Marketed since 1992 in Austria	
		1993 in Germany
		1999 in Japan and 34 other countries
**SP-2 (TachoComb H)**	Marketed since 2001 in Germany	
		2002 in Austria
**SP-3 (Tachosil®)**	EU Approval in 2004	

TachoSil® is indicated in adults as a supportive treatment in surgery for the improvement of hemostasis, to promote tissue sealing, and for suture support in vascular surgery where standard techniques are insufficient.

In many surgeries (digestive surgery, renal surgery, thoracic surgery, gynaecological surgery), tissue sealing involves the application of an agent, providing a seal and tissue sealing in addition to its action on the bleeding in the resection procedure. Tissue sealing agents consolidate the repair of organs, but also allow to fill the gaps generated by wide dissections and peri-organ detachments. Hemostatic and joining properties avoid disseminated or major bleeding, but also prevent the stagnation of fluid collections which can cause postoperative complications (pain, fever, peritonitis), and help reduce additional costs caused by a longer hospital stay.

At the present time, surgeons have no specific guidelines for tissue sealing. The consequences are inconsistent behaviours depending on the type of surgery and surgical department habits. Therefore, the criteria of choice in using of tissue collage by drug sponge remain unclear in terms of patient eligibility, types of surgery, application sites, etc.

The ELITE survey was designed to answer these questions and was approved by the French ethical committees CNOM ^a^, CCTIRS ^b^ and CNIL ^c^ in 2009.

## Methods

### Population

52 French surgeons participated in the ELITE survey during the year 2010 in the following surgical areas: abdominal, thoracic, gynaecology and urology. They recorded information on 683 operations performed from January to October 2010 where TachoSil® was used. The distribution in terms of surgical specialties was:

- Abdominal 63%
- Thoracic 14%
- Gynaecology 14%
- Urology 9%.

### Data collection

The following information was collected on paper CRFs:

- Demographic characteristics and medical history of patients at the time of inclusion.
- Pre-operative characteristics: Diagnosis, exams, preparation.
- Surgery: Anaesthesia type, surgery type, type and description of lesions, type of resection, dissection, anastomosis, etc.
- Bleeding risk and blood transfusions.
- Used of a medical sponge in terms of hemostasis techniques, application sites, number and size, and results in terms of success/failure.
- Patient follow-up in terms of outcomes, length of hospital stay, complications, death.
- Surgeon’s opinion regarding the objectives and reasons for choosing a medicated sponge.

### Plan for analysis

In order to investigate the criteria of use of TachoSil®, we extracted information from ELITE, focused on the following aspects:

- Type of lesions
- Type of procedures, including type of dissections
- Application sites characteristics.

The goal was to identify groups based on these aspects regardless the therapeutic area. The strategy was to transform these categorical or ordinal variables using a multiple correspondence analysis (MCA) to obtain independent numeric variables which could be used in an ascending hierarchical classification (AHC) using the Ward’s minimum variance method in order to obtain homogeneous clusters of patients regarding the selected criteria.

### Statistics

**Figure 1:**
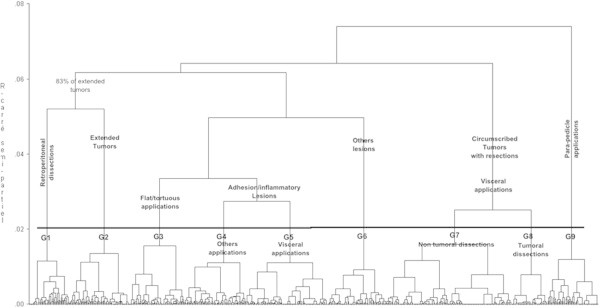
**Dendrogram illustrating cluster analysis results in 683 patients.** Patients were classified using agglomerative hierarchical cluster analysis based on axes from a multiple correspondence analysis. The statistical criteria left a choice between 6 or 9 clusters. Using 9 clusters led to a more specific characterization. The horizontal line identifies possible cut-off levels in the data for choosing the optimal number of groups.

Analyses were performed using the SAS® 9.2 statistical software. The data are presented as median values [interquartile range (IRQ)] or in percentage terms. The MCA was performed with the CORRESP proc, and the AHC with the CLUSTER proc, from SAS®. The TREE proc produced the dendrogram graphic (tree diagram) (Figure 1).

## Results

The demographic and medical history of the 683 patients is provided (see Additional file 1: Table S1).

### Group formation

18 categorical variables (binary or ordinal) were selected for their relevance to the lesions and the characterization of the application sites: 4 variables regarding the lesions, 10 variables for the type of procedure, including 7 types of dissections, and 4 variables describing the type of area applications (see Additional file 2: Table S2).

Using these variables, the MCA generated the Burt table, a symmetric table which shows the frequencies for all combinations of pairs of variables. The MCA led to 19 axes representing independent numeric variables. The coordinates of the 683 patients on these axes were used in an AHC, which is an agglomerative hierarchical clustering procedure. Each observation begins in a cluster by itself. The two closest clusters are then merged to form a new cluster, which in turn replaces the two former clusters. This merging of the two closest clusters is repeated until only one cluster remains.

The Ward’s Minimum-variance clustering method was used and the output resulted in a dendrogram (see Figure 1) showing the progressive joining of the clustering process from the 683 subjects (bottom) to one group (top).

Table 2 describes the last 15 steps of the clustering process. The pseudo F (PSF), and t^2^ (PST2) statistics are useful for estimating the number of clusters in the data. Relatively large values of PSF indicate good numbers of clusters. To interpret the values of the pseudo t^2^ statistic, navigate down the column until you find the first value that is markedly larger than the previous value, then move back up the column by one step in the cluster history. Considered together, these statistics suggest that the data can be grouped into 9 clusters, or 6 clusters in this study.

**Table 2:** **Last 15 generations of cluster history**

Cluster history										
NCL	Clusters joined		FREQ	SPRS	RSQ	ERSQ	CCC	PSF	PST2	Tie
**15**	CL29	CL47	48	0.0112	.467	.371	31.8	41.8	10.6	
**14**	CL119	CL25	48	0.0114	.456	.363	30.8	43.1	9.2	
**13**	CL26	CL40	75	0.0115	.444	.355	28.3	44.6	20.7	
**12**	CL28	CL30	61	0.0119	.432	.347	27.6	46.4	11.9	
**11**	CL15	CL55	71	0.0135	.419	.337	26.6	48.4	13.2	
**10**	CL21	CL42	67	0.0155	.403	.327	25.4	50.5	18.3	
**9**	CL13	CL22	151	0.0159	.387	.316	23.0	**53.3**	**28.7**	
**8**	CL9	CL23	194	0.0251	.362	.303	18.3	54.8	**40.3**	
**7**	CL17	CL16	172	0.0274	.335	.287	14.0	56.7	32.5	
**6**	CL10	CL7	239	0.0335	.301	.269	9.18	**58.4**	**32.9**	
**5**	CL6	CL19	309	0.0496	.252	.245	1.76	57.0	**46.1**	
**4**	CL14	CL11	119	0.0520	.200	.213	−3.5	56.5	39.9	
**3**	CL4	CL5	428	0.0617	.138	.166	−7.3	54.5	44.9	
**2**	CL3	CL8	622	0.0641	.074	.095	−7.1	54.4	50.3	
**1**	CL2	CL12	683	0.0740	.000	.000	0.00	.	54.4	

Based on the statistical criteria (pseudo F and pseudo t^2^ statistics) and a visual assessment of the dendrogram, an optimal number of 9 groups of patients (Figure 1) can be identified.

### Characterization of the 9 groups

From an overall point of view, the following table contains the main characteristic of each cluster (or group of clusters) read from the left to the right of the dendrogram (on Figure 1), with its percentage from the whole population (683). There is one group (G1) linked with retro-peritoneal dissections, and two groups (G3 and G9) linked with application site characteristics. All the others are based on the type of lesion.

Details of the groups are presented in Table 3, which shows the distribution of the different variables among them. Using this table, the characterization of each group can be refined:

- **G1 (7%)** includes only retroperitoneal large dissections mostly with circumscribed tumours in abdominal and urologic surgery on all kinds of application sites, excepted para-pedicle sites, which are almost all in the G9 group.
- **G2 (10%)** includes exclusively extended tumours (73% of the extended tumours are in this group) with enlarged resection (77%) and large dissections (82%), mostly parietal (58%). The application site is mostly visceral (62%).
- **G3 (10%)**: This group includes mainly operations using a medicated sponge on flat/tortuous sites (96%) related to circumscribed tumours or adhesion/inflammatory lesions with large dissections (99%), mostly visceral (77%).
- The next groups, **G4 (11%)** and **G5 (14%)** belong to the same branch related to tissue sealing adhesion/inflammatory lesions (respectively 69 and 85%), with visceral dissections. **G4** contains the highest rate of anastomosis (36%) and 84% of other application sites. **G5** involves 80% of abdominal surgery, only on visceral slice site.
- **G6 (10%)** contains exclusively lesions other than cancerous or inflammatory (100%), with mainly visceral dissections (70%). This also involves non pathological lesions due to various traumatic injuries. There is no specific application site. This group also has the highest rate of blood transfusions (37%).
- The next groups, **G7 (22%)** and **G8 (6%)**, belong to the same branch which involves 56% of circumscribed tumours resections on a visceral slice site of application. **G7** has no tumoral dissection and does not apply to urology. **G8** has procedures with 100% of tumoral dissection, mostly in urology.
- **G9 (9%)** appears as completely separated from the others in one specific branch. It nearly exclusively involves procedures with para-pedicle applications (98% of the group - 94% of the para-pedicle applications), involving all kinds of lesions with mostly large lymphatic dissections (64%).

**Table 3:** **Description of the profiles of the 9 identified clusters**

	G 1	G 2	G 3	G 4	G 5	G 6	G 7	G 8	G 9
	N = 48 (7%)	N = 71 (10%)	N = 67 (10%)	N = 74 (11%)	N = 98 (14%)	N = 70 (10%)	N = 151 (22%)	N = 43 (6%)	N = 61 (9%)
Circumscribed tumour	**60**	7	63	35	20	4	**97 (43)**	**100 (13)**	46
Extended tumour	21	**100 (73)**	1	4	2	0	1	0	16
Adhesion/inflammatory	10	10	46	**69**	**85**	10	7	2	33
Other lesions	21	1	1	0	2	**100 (76)**	0	0	13
Resection	79	**90**	76	81	63	39	**100**	**100**	89
Resection size									
0 (none) to 2	27	11	24	22	46	67	2	0	15
2–5 cm	27	17	6	8	16	9	23	53	5
>5 cm	46	**72**	70	70	38	24	**75**	47	**80**
Anastomosis	31	**32**	28	**36**	12	13	11	16	18
Enlarged resection	44	**77**	46	36	7	9	27	12	30
Dissection									
- Parietal	17	**58**	34	30	11	21	40	12	15
- Visceral	46	52	**70**	**70**	**69**	**70**	**62**	42	30
- Adhesion	19	35	43	58	53	22	17	7	20
- Pedicle	23	23	13	26	23	14	19	30	**77**
- Tumoral	15	32	13	4	6	3	0	**100 (42)**	12
- Lymphatic	23	32	28	12	0	0	22	0	**64 (29)**
- Retro-peritoneal	**100 (96)**	0	1	0	0	0	0	0	2
Dissection size									
0–2 cm	2	0	0	0	7	5	1	0	0
2–5 cm	6	18	1	13	29	17	20	44	8
>5 cm	**92**	82	**99**	87	64	78	79	56	**92**
Application site									
- Visceral slice	50	62	22	22	**100**	57	**97**	**98**	7
- Flat/tortuous	40	35	**96 (46)**	5	1	26	1	0	10
- Para-pedicle	2	0	2	1	0	1	0	0	**98 (94)**
- Other	25	23	2	**84 (44)**	2	29	4	0	38
Surgery									
- Abdominal	50	48	73	65	80	67	58	44	74
- Thoracic	4	20	5	26	12	4	23	0	13
- Gynaecologic	6	25	13	8	5	**27**	19	2	7
- Urologic	**40 (30)**	7	9	1	3	1	1	**54 (36)**	7
Bleeding risk	27	24	30	31	23	**35**	24	12	31
Blood transfusion	19	19	18	19	8	**37**	13	7	20

Values represent % within each group.

Value between parenthesis represent % in the whole population (N = 683).

The large sponge type was used in 81% of the cases, covering an area of about 5 cm^2^ in 50% of cases, regardless of the type of surgery. The areas were located in the parenchyma, flat areas, and also the anfractuous pedicle.

## Discussion

A good hemostasis, enhanced by the use of hemostatic agents when necessary, is important in reducing the risk of intra-surgical and post-surgical complications. This is the case of digestive leakage in hepato-biliary and pancreas surgery, the development of a lymphocele after a lymphadenectomy, or postsurgical adhesions. These complications weigh heavily on operated patients morbidity and lead to additional healthcare cost (Lasser 2006;Johns 2001).

TachoSil® is indicated in adults for supportive treatment in surgery for improvement of haemostasis, to promote tissue sealing, and for suture support in vascular surgery where standard techniques are insufficient (SmPC TachoSil® 2013).

Table 3 shows the distribution of lesion sites within the 9 groups. This information was recorded in the ELITE database but not used in the group formation process. This table is helpful for refining the identification of the groups.

The G1 group (7%) includes only retroperitoneal large dissections in abdominal (50%) and renal surgery (40%) on all kinds of application sites. The surgery concerned is located in the pancreas and at renal level, and TachoSil® is often applied to these organs in its indication.

After each definition group of patients eligible for tissue sealing, one or more clinical trials showing the application of TachoSil® in the anatomical area concerned.

Padillo et al. (2010) analyzed the incidence of intra-abdominal infectious complications after the application of TachoSil® to the duodenojejunal anastomosis in simultaneous pancreas–kidney transplants (SPK) with enteric drainage. 68 SPKs with enteric drainage were prospectively assessed. TachoSil® was applied to the duodenojejunal anastomosis in 34 patients, who were compared to a control group of 34 patients. The incidence and severity of intra-abdominal infectious complications and the 1-year patient and graft survival rates were analyzed. Eighteen patients experienced intra-abdominal infectious complications. Grade 1a complications occurred in the study group, whereas surgery was required only in patients from the control group: grade 3a complications (15%) and grade 3b complications (18%) (p = 0.003 vs. study group, respectively). The overall rate of anastomotic leakage (grade 2b and 3b complications) was 10%, all of which occurred in the control group. The length of hospital stay was higher in the control group: 34.6 ± 11.3 days vs. 22.8 ± 11.1 days (p = 0.03). There were no significant differences in 1-year patient and graft survival rates between the groups.

The G2 group (10%), exclusively extended tumours, corresponds to large dissections during resection procedures related to abdominal surgeries (48%), but also to gynaecological procedures (25%) and thoracic surgeries (20%). These are wide-scale surgical resections, with 77% of enlarged resections and 58% of parietal resections. 78% of lymph node dissection belong to this group, with mostly pelvic and intra abdominal extensions. Maggiore et al. (2011) published a prospective, randomized trial in which they measured the application of TachoSil® after laparoscopic myomectomy due to intramural uterine myomas. 70 patients were randomized into the two groups. All surgical procedures were performed laparoscopically and no conversion to laparotomy was needed. There were no major surgical complications such as bowel, bladder or ureteral injuries. The operating time (p = 0.870) and length of hospital stay (p = 0.306) were similar in the two study groups. The median value of TachoSil® patches applied in the patients included in group A was 1 (range, 1–3); the mean time (±SD) required for TachoSil® application was 7.1 ± 3.4 minutes. Blood loss in group A was significantly lower than in group B (p = 0.005); no blood transfusion was needed in either group of patients. The drainage fluid collected was significantly lower in group A than in group B (p < 0.001). Fifteen patients in group A and 13 in group B tried to conceive 6 months after the surgery. Eight patients in group A (53.3%) and 3 in group B (23.1%) successfully conceived (p = 0.212).

The G3 group (10%) includes mainly operations using TachoSil® on flat/tortuous sites (96%) related to circumscribed tumours or adhesions/inflammatory lesions, involving large dissections (> 5 cm surface) (99%), mostly visceral (73%). This is the case in liver surgery, as shown by a large amount of data available in the literature.

Fischer et al. (2010) designed an international, multicenter, randomized, controlled surgical trial with 2 parallel groups. Patients were eligible for intra-operative randomization after an elective resection of ≥ 1 liver segment and primary hemostasis. The primary endpoint was the time to hemostasis after starting the randomized intervention in order to obtain secondary hemostasis. Secondary endpoints included drainage duration, volume, and content. Adverse events were collected to evaluate the safety of the treatments. Among the 119 patients (60 TachoSil® and 59 ABC) who were randomized in 10 tertiary care centres in Europe, the mean time to hemostasis was less when TachoSil® was used (3.6 minutes) compared with ABC (5.0 minutes; p = 0.0018). The estimated ratio of the mean time to hemostasis for TachoSil®/ABC was 0.61 (with a 95% confidence interval, 0.47–0.80; p = 0.0003).

The G4 (11%) and G5 groups (14%) describe situations of adhesions/inflammatory lesions (respectively 69% and 85%) and correspond to abdominal surgery (respectively 65% and 80%) and pulmonary surgery (respectively 26% and 12%). These cases involve situations of adjuvant hemostasis to promote tissue sealing in hepatobiliary surgery. 53% of gall bladder lesions are in group 5. In lung surgery, they are mostly situations where TachoSil® was used more as a haemostatic agent (31% of bleeding risk in the G4 group) than as a tissue sealing agent to treat air leakages (50% of pleura lesions belong to group 5).

In lung surgery, Marta et al. (2010) evaluated the efficacy and safety of TachoSil® versus a standard treatment of air leakage after lobectomy in a randomized, prospective, parallel-group trial.

A total of 486 patients were screened and 299 received the trial treatment (intent-to-treat (ITT) population: TachoSil®, n = 148; standard treatment, n = 151). TachoSil® resulted in a reduction in the duration of postoperative air leakage (p = 0.030). Patients in the TachoSil® group also experienced a greater reduction in intra-operative air leakage intensity (p = 0.042). Median time until chest drain removal was 4 days with TachoSil® and 5 days in the standard group (p = 0.054).

Briceno et al. (2010) conducted a prospective, controlled study with a total of 115 patients (58 in the control group and 57 in the TachoSil® group) scheduled for conventional hepatectomies. After a major liver resection, the TachoSil® group was effective for decreased drainage volume (mean [SD] volume: 1124.7 [842.8] mL in the control group and 691.2 [499.5] mL in the TachoSil® group; p = 0.007), with a higher volume of drain output on each postoperative day among the control-group patients (p = 0.003); for postoperative blood transfusion requirements (18.9% vs. 7.0%, respectively; p = 0.04); for moderate to severe postoperative complications (21% vs. 8%, respectively; p = 0.03); and for mean (SD) duration of hospital stay (12.6 [6.7] vs. 9.6[5.1] days, respectively; p = 0.03).

Two consecutive cohorts of 16 patients undergoing an adult right lobe split liver transplant were compared by Toti et al. (2009). In the first cohort, the liver surface was treated with fibrin glue, and in the second the liver surface was treated with TachoSil®. The post-operative complications were analyzed.

Bile leaks were significantly fewer among those patients in whom the cut surface of the liver was treated with TachoSil® compared to those for whom fibrin glue was used on the cut surface: 1/16 (6.25%) vs. 7/16 (43.75%), respectively; p = 0.03. There were some differences in the biliary anastomotic techniques used in the two groups, but 7 out of 8 leaks (87.5%) arose from the cut surface, and only one was from the anastomosis.

The G6 group (10%) contains non pathological lesions due to various traumatic injuries. This group also has the highest rate of blood transfusions (37%). TachoSil® has an elective indication in adjuvant hemostasis and has proven to be a powerful hemostasis agent.

Grottke et al. (2010) measured severe bleeding in a coagulopathic pig model with a blunt liver injury. Following surgical preparation, which included splenectomy and cystotomy, a coagulopathy was induced by exchanging 80% of the animals’ blood volume with hydroxyethyl starch 130/0.4 and lactated Ringer’s solution. Subsequently, a grade III liver injury was induced by a force of 238 ± 19 newtons and free bleeding was allowed for 30 s. The animals were randomly assigned to receive either a placebo patch (cotton patch) (control group, n = 7) or a fibrinogen/thrombin patch (TachoSil® group, n = 7), which was positioned 30 s after injury on the inflicted area. The coagulation parameters, hemodynamic variables, as well as the treatment were monitored for 2 h post-injury and patch placement. A histology was obtained to evaluate the equality of the liver injury and to show the morphology of the TachoSil®. Hemostasis after hemodilution was severely impaired. The amount of blood loss after the trauma was significantly diminished in the TachoSil® group (419 mL ± 90 mL) compared with the control group (1775 mL ± 358 mL) (p < 0.001). All the animals treated with the TachoSil® survived, whereas 100% of the control group died before reaching the end of the observation period (p < 0 .001).

TachoSil® has successfully been used in liver and spleen trauma (Rojnoveanu et al. 2010; Abu Hilal et al. 2007; Apestegui et al. 2009), and also in various situations of gynaecological haemorrhages (Bennich et al. 2008; Fuglsang and Petersen 2010; Shirata et al. 2007; Tekesin et al. 1999).

Group G7 group (22%) is composed of cases of non-tumour dissection in abdominal surgery (58%) (44% of liver lesions), thoracic surgery (23%) (42% of lung lesions), and gynaecology (19%) surgery (78% of breast lesions).

In Group G8 (6%), 100% of the procedures are in renal surgery.

Siemer et al. (2007) randomized a total of 185 patients scheduled for an elective nephron-sparing surgery for small, superficial kidney tumours included in an open, randomized, prospective, multicenter, parallel-group trial. The primary objectives were to test the haemostatic efficacy and safety of TachoSil® versus standard suturing. Efficacy was tested by comparing intraoperative time to hemostasis (primary endpoint). The secondary objectives included determining the proportion of subjects with hemostasis after 10 min of trial treatment, the occurrence of a hematoma on day 2 after surgery, the volume and haemoglobin concentration of postoperative drainage fluid, and the surgeon’s rating of the usefulness of the trial treatments. Safety was evaluated based on the occurrence of adverse events. In the intent-to-treat population, the time to hemostasis was significantly shorter with TachoSil® versus standard suturing (mean: 5.3 vs. 9.5 min [p < 0.0001]). Hemostasis was obtained within 10 min in 92% of patients in the TachoSil® group and in 67% in the standard treatment group (p < 0.0001).

TachoSil® has an excellent indication for adjuvant hemostasis in partial nephrectomy by open or laparoscopic or robotic surgery (Fuglsang and Petersen 2010; Hama Attar et al. 2008; Sanseverino et al. 2009). Ready-to-use and easy to apply on the kidney, it avoids the extra time of renal ischemia (Simone et al. 2009; Papalia et al. 2009).

Group G9 (9%) involves almost exclusively procedures with para-pedicle applications related to all kinds of lesions, with mostly large lymphatic dissections (64%). 74% of thyroid problems belong to this group.

In cancer surgery, haemostatic agents are used to prevent lymphorrhea. The aim is to occlude the smaller lymphatic channels, knowing that lymph has a composition close to the blood coagulation factors secreted by the lymphatic endothelial cells, but its coagulation is much slower than that of blood. The addition of local hemostatics can accelerate lymph coagulation. TachoSil® contains fibrinogen and thrombin as a dried coating on the surface of the collagen sponge. When in contact with physiological fluids, e.g. blood, lymph, or physiological saline solution, the components of the coating dissolve and partly spread over the wound surface (SmPC TachoSil® 2013).

A total of 60 consecutive patients who had undergone radical prostatectomy and pelvic lymphadenectomy were prospectively enrolled in this study. The patients were randomly assigned to a standard technique with the use of clips and electrocoagulation plus TachoSil®, or to standard technique only. All patients underwent ultrasound examination on postoperative days 7, 14 and 28 to test for the development of symptomatic or asymptomatic lymphoceles. Drainage volume and duration were also recorded. The baseline characteristics of the 2 randomized groups were well matched. Those patients in whom we used TachoSil® showed a lower drainage volume with a mean total volume of 64 ± 45 ml (range 0 to 110) vs. 190 ± 62.72 ml (range 70 to 270, p = 0.009), and had significantly fewer symptomatic and asymptomatic lymphoceles (5 vs. 19, p = 0.001) (Simonato et al. 2009).

Ghelardi et al. (2011) compared two groups, each consisting of 11 women submitted to inguinofemoral lymphadenectomy during surgery for invasive vulvar cancer. The experimental group was treated with TachoSil®, a collagen-based product coated with fibrinogen and thrombin, and was compared with a control group in which TachoSil® was not used. We analyzed the data on fluid drainage considering the total amount collected in the observation period and the peak values. The group treated with TachoSil® had less lymphorrhea, recording a mean total volume of 635.5 ml, vs. 833.6 ml in the control group (p <0.001). The peak volumes in the TachoSil® and the control groups were, respectively, 148.6 ml vs. 193.6 ml (p = 0.019).

The bleeding risk was also evaluated according to the G1-G9 groups, and it appeared higher in the G6 group (35%) of abdominal (67%) and gynaecological surgeries (27%), in the G4 group (31%) composed of abdominal (65%) and thoracic surgeries (26%), and in the G3 group (30%) corresponding to abdominal (73%) and gynaecological (13%) surgeries. Blood transfusion was also significant in the G6 group (37%).

## Conclusion

TachoSil® is the only ready-to-use fixed combination of human fibrinogen and thrombin coated onto a collagen patch. Its indication and pharmaco-mechanical properties allow it to ensure effective hemostasis and tissue sealing in various surgeries.

## Endnotes

^a^CNOM: National Council of the College of Physicians.

^b^CCTIRS: Advisory Committee on Information processing Research in the field of Health.

^c^CNIL: National Commission of Information and Liberties.

## Electronic supplementary material

Additional file 1: Table S1: Patient characteristics. (DOC 32 KB)

Additional file 2: Table S2: Variables used to build the model. (DOC 34 KB)
