# Supplementary material for: Patient eligibility criteria for a surgical treatment that enhances tissue sealing by use of a medicated sponge: observational study ELITE
Source: Springerplus. 2013 Nov 18;2:613. doi: 10.1186/2193-1801-2-613 (PMC3847034; doi:10.1186/2193-1801-2-613)
Supplement: Supplementary file 1 — Additional file 1: Table S1: Patient characteristics. (DOC 32 KB) [file 40064_2013_668_MOESM1_ESM.doc]

**Table** S1: Patient characteristics

| **Patients N=683** | |
| --- | --- |
| **Variable** | **Result** |
| **Sex** | M 52% W 48% |
| **Age, years** | 61 [47 – 72] |
| **Weight, kg** | 72 [62 – 82] |
| **Height, cm** | 168 [162 – 173] |
| **Previous surgery, Y/N** | Yes 65% |
| **Current operation** |  |
| - **Abdominal** | 63.1% |
| - **Urology** | 9.4% |
| - **Gynaecology** | 13.6% |
| - **Thoracic** | 13.9% |
| **Bleeding risk** | Yes: 26% |

Numeric variables are presented as median values [Q1 – Q3]
