# Supplementary material for: Patient eligibility criteria for a surgical treatment that enhances tissue sealing by use of a medicated sponge: observational study ELITE
Source: Springerplus. 2013 Nov 18;2:613. doi: 10.1186/2193-1801-2-613 (PMC3847034; doi:10.1186/2193-1801-2-613)
Supplement: Supplementary file 2 — Additional file 2: Table S2: Variables used to build the model. (DOC 34 KB) [file 40064_2013_668_MOESM2_ESM.doc]

**Table** S2: Variables used to build the model

| **Variable**  **Number** | **Parameter** | **Number (%)** |
| --- | --- | --- |
| **Type of lesion** | | |
| **1** | **Circumscribed Tumour** | 343 (50.2) |
| **2** | **Extended Tumour** | 98 (14.4) |
| **3** | **Adhesion/inflammatory** | 215 (31.5) |
| **4** | **Other** | 92 (13.5) |
| **Type of operation** | | |
| **5** | **Resection size**  **- 0 (none) - 2 cm**  **- 2 – 5 cm**  **- > 5 cm** | 157 (23.0)  117 (17.1)  409 (60.0) |
| **6** | **Anastomosis** | 139 (20.4) |
| **7** | **Enlarged resection** | 211 (30.9) |
| **8**  **9**  **10**  **11**  **12**  **13**  **14** | **Dissection**  **- Parietal**  **- Visceral**  **- Adhesion**  **- Pedicle**  **- Tumoral**  **- Lymphatic**  **- Retro-peritoneal** | 195 (28.6)  405 (59.3)  215 (31.5)  176 (25.8)  102 (14.9)  134 (19.6)  50 (7.3) |
| **Application site** | | |
| **15** | **Visceral slice** | 430 (63.0) |
| **16** | **Flat/Tortuous** | 139 (20.4) |
| **17** | **Para-pedicle** | 64 (9.4) |
| **18** | **Other** | 142 (20.8) |
